# Emerging role of pioneer transcription factors in targeted ERα positive breast cancer

**DOI:** 10.37349/etat.2021.00031

**Published:** 2021-02-28

**Authors:** Honey Pavithran, Ranjith Kumavath

**Affiliations:** Department of Genomic Science, School of Biological Sciences, Central University of Kerala, Tejaswini Hills, Periya (PO), Kasaragod, Kerala 671320, India; National University of Singapore, Singapore

**Keywords:** Transcription factor, pioneer transcription factors, ERα signaling, breast cancer

## Abstract

Transcription factors (TFs) are modular protein groups that preferably bind to DNA sequences and guide genomic expression through transcription. Among these key regulators, “pioneer factors” are an emerging class of TFs that specifically interact with nucleosomal DNA and facilitate accessible genomic binding sites for the additional TFs. There is growing evidence of these specialized modulators in particular malignancies, as highlighted by agents’ clinical efficacy, specifically targeting nuclear hormone receptors. They have been implicated in multiple cancers more recently, with a high proportion inculpating on hormone influential cancers. Moreover, extended crosstalk and cooperation between ERα pioneering factors in estrogen-dependent breast cancer (BC) remain elucidated. This review discusses on the recent advances in our understanding of pioneer TFs in cancer, especially highlighting its potentiality to modulate chromatin condensation to permit ERα recruitment in BC cells. Through the study it was concluded that the highly prospected pioneer TFs in BC, including FOXA1, TLE1, PBX1, and GATA3, possess the potential therapeutic significance and further innovations in the field could yield targeted therapy in cancer treatment.

## Introduction

Transcription factors (TFs) include the most prominent regulatory proteins that bind to the specific region in the DNA and control gene expression by influencing RNA polymerase activity [1, 2]. DNA sequences of 6–20 bp within the transcriptional initiation sites referred to as TF binding sites are recognized by these specialized proteins and regulate the transcription of genes [3]. The biological diversity among these proteins is striking; they range from a smaller group of general TFs to many proteins called regulatory TFs [4]. Besides, an emerging subset of unique TFs termed pioneer transcription factors (PTFs) that specifically bind to allocated enhancers in the nucleosomal DNA and facilitates accessible genomic binding sites for additional TFs intimated with transcriptional activity [5]. PTFs are specialized TFs that can autonomously bind to compact chromatin with the peculiarities of overcoming constraints and assisting the binding of non-pioneer TFs [6, 7]. As the name suggests, pioneer factors are the prior members to approach and engage themselves to the chromatin binding sites [8]. Discrete protein families exhibit pioneer factors’ specialized properties, including forkhead box protein A1 (FOXA1), TLE, and GATA family. Moreover, they are associated with several other roles, including cell fate in the developmental process, cell reprogramming, and positively insisted on various types of cancer imprinting high generosity in hormone-dependent solid tumors [5].

Estrogen receptor (ER) is a member of the nuclear receptor that acts as a proliferative TF and drive tumorigenesis in mammary epithelial cells. It is evident for a very long that in ER^+^ breast cancer (BC), the ER cooperatively interacts with other TFs, co-factor, and pioneer factors and causes malignancy. This review is intended to share our superior understanding regarding pioneer factors in the context of BC. The mechanistic role executed by PTF in recruiting estrogen receptor α (ERα) receptors on the genomic DNA and thus imparting its indispensable role in ERα signaling regulation in BC is explored through comprehensive literature analysis. ER also functions as part of a sizeable transcriptional complex involving multiple TFs, including its pioneer factor, FOXA1, GATA binding protein 3 (GATA3), pre-B-cell leukemia transcription factor 1 (PBX1), and transducin-like enhancer protein 1 (TLE1). Many of these modulate the ER pathway activity by directly affecting the binding of ER to chromatin [9]. Some of the well-explored signaling components in transcriptional machinery, such as the family of nuclear factor kappa-light-chain-enhancer of activated B cells (NF-κB) proteins is a key signaling molecule in several human diseases, including cancers directly or indirectly influenced by the activity of PTF in the signal cascade system in various cancers [10]. The current view discussed in detail through the review is that the recruitment of PTF to chromatin enables the protein to scan the surrounding sequence further and provide accession to the downstream targets in normal and dysregulated context.

## ER dependence on BC

BC is a highly heterogeneous disease with over 2.1 million new diagnoses every year and transpired to be the second leading cause of death from cancer in women worldwide [11, 12]. The advent of high throughput gene expression profiling (GEP) such as microarrays has led to a new standard in interpreting the heterogeneity within the disease based on which breast tumors were classified into five intrinsic subtypes including luminal A, luminal B, HER-2 over-expression, basal and normal-like tumors [13]. Among these five intrinsic groups’ luminal tumors that express ER and responsive genes are accounted to be the most common subtype, representing about 75% of patients diagnosed with BC [14].

ERs are ligand-gated TFs that regulate several key biological processes, including estrogens’ effects at gene regulation level [15]. The two significant isoforms of the ER, ERα and estrogen receptor β (ERβ), belong to the nuclear hormone receptor (NHR) superfamily of TFs [16]. ERα is regarded as the master transcriptional regulator in the mammary gland development and the driving dysregulated factor in BC malignancy [17]. The estrogen hormone’s genomic expression is largely mediated through ERα receptor, and there exist two well-explored pathways that brief about the receptor’s gene regulatory function. According to the molecular classification of estrogen, the action involves estrogen binding to ERα in the cytoplasm followed by receptor dimerization. Then, the ligand-receptor complex translocates to the nucleus and binds to estrogen response elements (ERE) located on the targeted gene’s genomic DNA. This occurs through a protein-protein interaction with the association of other TFs [18]. Whereas the second signaling pathway, referred to as the classical non-mechanism, is controlled by an indirect ER binding to DNA, with the aid of several co-factors (SP-1, AP-1, and NF-κB) that regulate gene transcription [19].

It is evident that BC patients diagnosed with ER-positive and progesterone receptor-positive have a lower risk of mortality and are treated with adjuvant hormonal or chemotherapeutic regimens. However, BC-specific mortality risks were elevated among women with ER^+^/PR^−^, ER^−^/PR^+^, and ER^−^/PR^−^ tumors relative to women with ER^+^/PR^+^ tumors across all subcategories of age cancer diagnosis [20, 21]. Contradict to this scenario, in a study on the effects of ER status and other prognostic factors, including *BRCA2* mutation analysis, positive ER status was associated with a higher risk of death than negative ER status on further confirming that ER status is an adverse prognostic factor [22]. Furthermore, the mutation in ER gene *ESR1* has recently been recognized with resistance to estrogen deprivation and relative resistance to tamoxifen and fulvestrant [23, 24]. Therefore targeting ERα for degradation is an effective treatment in too severe metastatic ER-positive BC stages. A recent study on the prospect of ER degradation showed that proteolysis-targeting chimera (PROTAC) technology could be an emerging paradigm for protein degradation, which can eliminate both wild type and mutant ERα in BC cells [25]. Another emerging class of multifunctional TF CTCF is a ubiquitous multivalent zinc finger protein that involves transcriptional machinery and regulates higher-order chromatin organization [26]. The proteins are known to regulate gene expression through various mechanisms, including recruiting other co-activators and binding them to promoter regions of target genes [27]. Recent advanced studies report that CTCF binding can co-localize with ER and FOXA1 binding and shown to be required for hormone-responsive silencing of target genes, together with the nuclear receptors, thyroid hormone receptor, and retinoic acid receptor [28].

### The essential role of pioneer factors in ERα dependent BC

**Figure 1.**
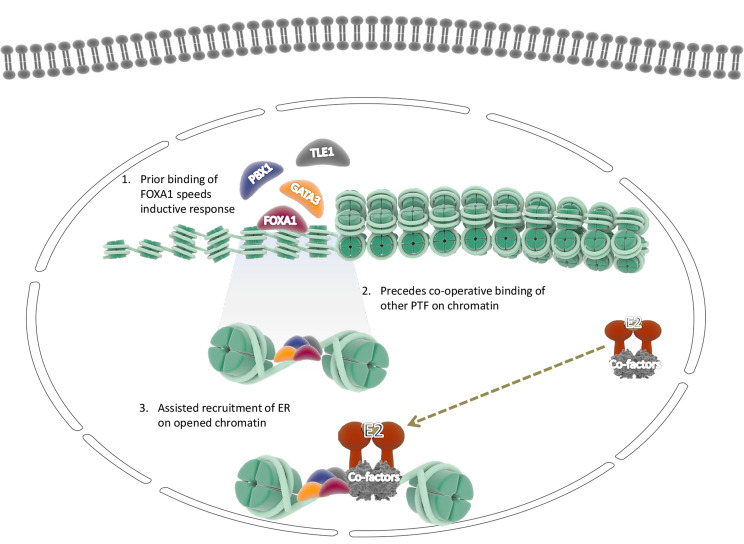
An alternative view of pioneer TFs involved in ERα recruitment on the chromatin further facilitates the assisted loading of induced dimers of ER and promotes ER expression followed by the induction of downstream genes. The pioneer factors gain access to regulatory elements in a sequential manner through cooperative functioning between pioneer factors, TFs, and co-factors (1) FOXA1 pioneer factor binds to the specific motif in the chromatin (2) followed by the cooperative binding of other factors to their respective motif (3). This effectively promotes chromatin’s de-compactness and further leads to the recruitment of estrogen hormone-induced ER dimers on chromatin, which programs the ER dependent genes and proceeds transcription. E2: ubiquitin-conjugating enzyme

The role of activated ER and the associated proteins that help it tether to the genomic DNA is explicitly becoming well documented in BC. Thus, a growing number of studies are carried out to characterize chromatin accessibility by assessing active status. TFs access chromatin to bind to their enhancer elements and thus regulate gene expression. Therefore, it is evident that ER requires the cooperative action of multiple factors to gain access to binding sites in the closed chromatin state through direct or indirect influences. The biological activity of estrogen in the mammary gland’s epithelial cells is primarily mediated through binding and activating the ERα factor. Subsequently, the activated ERα binds to genomic DNA with numerous other proteins, including pioneer factor facilitating a permissive state of gene expression. Besides, steroid receptors such as ER and androgen receptor (AR) exhibit pioneer properties in a chromatin dependent manner. The usual context executes its non-pioneer role via involvement in the regulation transcription machinery devoid of pioneer activity [29]. The use of highly sophisticated TF mapping techniques equipped to investigate ER-associated proteins and to understand the chromatin competence that facilitated ER binding to chromatin explored a wide range of ER-dependent pioneer TFs [8]. Our study collectively endeavored to shed light on the versatile mechanisms used by pioneer factors to engage their target sites in closed chromatin. Initial studies in pioneer transcriptional regulation reported that FOXA1 acts as a pioneer factor for binding the ER in BC cells and played a pivotal central role in activating the gene. Recently more outstanding works in association with ER and pioneer function in chromatin regulation further explored other PTF such as GATA3, TLE1, and PBX1, which influenced chromatin binding and was necessary for ER binding (Figure 1).

## Pioneer factors involved in ERα recruitment on the genome

FOXA1, is a prominent pioneer factor and the benefited prior member to bind to the chromatin domain for further enabling gene activity [30]. They independently bind to and de-compacts condensed chromatin to facilitate the binding of other TFs such as ER and AR [31]. Accordingly, recent findings point towards the significant impact of FOXA1 in modulating nuclear steroid receptor activity in breast and prostate cancer, suggesting that FOXA1 may significantly contribute to pro-tumorigenic phenotype [32, 33]. The importance of FOXA1 regulation of prostatic and non-prostatic AR-chromatin targeting is well implicated through several studies and also reported as the third most frequently mutated gene in prostate cancer [21]. In the follow-up study of hormone-dependent cancers and pioneer factors, Jozwik and Carroll [5] reported that FOXA1 bounds about 50% of all ER binding sites. The study also inferred a strong correlation between FOXA1 and ER, which further validated its strong influence in ER-dependent BC. Moreover, a recent study encompassing genomic analysis of FOXA1 associated with hormonal cancers showed that FOXA1 binding events are not regulated by hormones [34].

GATA3 belongs to the zinc-finger TF family, a vital aliment concerned with luminal epithelial cell differentiation and commitment, and is a strong predictor of clinical outcome in humans luminal BC [35]. Thus reports suggest that 15% of ER-positive BC shows *GATA3* mutation together with other transcriptional deficiency [36]. GATA3 binds to the chromatin and plays a the pioneering role in the recruitment of ERα and promotes the abnormal proliferation of cells as well as it strongly correlates with ER expression in BC malignancy [37]. GATA3 functions with FOXA1 and ER to enhance ER-responsive genes in normal and pathological situations [38]. Studies provide evidence of additional ERα-cooperating activity of GATA3, which can mediate with enhancer at ERα regulatory regions and has properties similar to FOXA1 [39, 40]. Here we portray the strong evidence of GATA3 proteins in association with ERα recruitment and BC occurrences.

TLE1, also known as the human Groucho protein, is required for ER binding and is mentioned to be an introductory class of factors fundamentally required for ER-mediated gene transcription [5]. It was demonstrated that TLE1 exhibit multitasking property by positively assisting ER-chromatin interaction being a transcriptional repressor. Furthermore, specific silencing of TLE1 inhibits the ability of ER to bind to its binding site in the genome. The utmost concern factor-related TLE1 is its efficacy in ER-mediated cell division [41].

PBX1 is an important member of the PBX family of proteins, the suspected group of factors in various cancers. A very recent study incorporating expressional patterns of the PBX family in association with BC revealed that among all PBX family members, PBX1 was only significantly upregulated in BC [42]. GATA family of proteins include GATA3 and GATA4 protein, which are deregulated in various cancers. Studies lead to identifying the expressional role of GATA3 in association with breast cells identified its involvement in mammary gland morphogenesis, particularly in luminal cell differentiation [43].

The mutation landscape of TFs that adversely affect human cancers is widely studied and addressed in an astonishing number of findings that support therapeutic tweak from the current treatment stage provided in BC. Thus, a detailed exploration of pioneer TFs studying their gene expression patterns across BCis elaborated through our study (Table 1).

**Table 1.** Predicted pioneer TFs in ERα dependent BC

**Pioneer factors**	**Molecular/cellular context**	**Pioneer activity**	**References**
FOXA1	Mitotic biomarker in human hormone-dependent cancer progression	Chromatin decompaction through binding to mitotic chromatin and recruitment of nuclear receptors	[33, 34]
TLE1	Ubiquitous expression in the endometrium, and immunohistochemical biomarker in breast carcinogenesis and synovial sarcoma diagnosis	Multitasking transcriptional corepressor that associate with condensed chromatin by binding to the histone tails of nucleosomes	[44, 45]
PBX1	Specific biomarkers for the diagnosis and prognosis of BC, prostate cancer, and an evident therapeutic target	Translates epigenetic cues and mediates estrogen-induced ERα binding	[28, 46]
AP2γ	The determinant of helix-span-helix domain and its binding affinity to DNA as a dimer is based on the net basic charge, the functional discrepancy of HS-1 hypersensitivity in ER-dependent cancer	Capable of transactivating human ERα promoter in BC through high-affinity AP2 sites in the untranslated leader sequence	[47, 48]
GATA3	Associated with endodermal development	Binds to nucleosomal DNA and actively promote decompaction of chromatin, induce nucleosome eviction	[49]
GATA4	Functionally associated with endodermal development	Capable of binding to compacted chromatin and to open the local nucleosome-rich domains, even in the absence of ATP-dependent chromatin remodeling enzymes, cooperative activity between FOXA1 in chromatin binding	[50, 51]

The genomic region inculpating the *FOXA1* gene is amplified in different cancers with the prior evidence collected from prostate and BC [52]. An experimental analysis carried out by Gan et al. [53] on the emerging role of PTF and liver cancer reported that silencing of *FOXA1* gene regulates liver cancer progression through suppressing cancer stem cell proliferation and apoptosis machinery. GATA3 is another well-characterized PTF that influences immune cell function. The ectopic expression of GATA3 in human 293T cells caused the induction of 73 genes, including six cytokeratins, and inhibited cell line doubling times. These findings together strongly support the role of GATA3 in growth control, and variants may contribute to tumorigenesis in ER-positive BC [54].

A comprehensive study on genetic alterations and targeted therapies in acute myeloid leukemia portrayed the importance of epigenetic gene mutation and suggested the persistence of TFs and prognostic and therapeutic efficacy in several human malignancies, including cancer [55, 56]. In many human cancers, the mutation in upstream regulators and aberrant gene amplification destabilize the TF network’s proper functioning. Accordingly, there is a need for tools to intervene directly with TFs for exploiting these targets therapeutically. Small molecule intervention is a crucial avenue in addressing deregulation in transcriptional machinery, providing complex feedback and regulatory mechanism that works in a healthy context [57, 58].

## Therapeutic significance of targeting ER-associated pioneer factors in BC

ER status is an important criterion undertaken during prognosis and treatment selection in BC patients [59]. However, reports suggest that annually 20–40% of patients medicated for BC eventually develop metastasis, and this phenomenon is more pronounced in ER-positive BC [60]. Another critical discrepancy related to ER-mediated BC is drug resistance to approved systematic therapies [61]. Well hypothesized studies on the association of drug resistance and BC points out several factors, including micro-environmental agents, growth factor-mediated, ER influenced signaling, and molecular aberrations to this cause [62]. An outcome focusing on the underlying molecular perspective in ER^+^ BC identifies transcriptional activity-driven cell proliferation from the research sets. It provides evidence from transcriptional regulators, pioneer factors in mediating ER-chromatin interactions [8]. Several groups of scientific studies targeting transcriptional activity once purported that ER could initiate gene transcription, hence making the ligand (estrogen) and receptor (ER) the sole determinants of its activity [63].

There is an emerging class of TFs, the pioneer factors receiving esteemed importance in therapeutics and clinical efficacy. The active involvement of pioneer factors such as FOXA1, GATA3, TLE1, and PBX1 enables ERα chromatin binding and, thus, crucial for ERα has driven cell proliferation in BC [64]. From the class, the imperative being widely researched is FOXA1, as it is an essential determinant of ER function even in an endocrine-resistant context [65]. Yamaguchi et al. [66] showed that chromatin immunoprecipitation (ChIP) in tamoxifen-resistant MCF-7 cells that reduce FOXA1 expression induced IL6 expression and eventually contributed cancerous properties and can be targeted in ER-positive BC. A recent comprehensive genome-wide study of *FOXA1* mutation in ER-positive BC illustrated the adequate chromatin accessibility, localization on chromatin, and the transcription effect on mutant *FOXA1* by imparting its role in driving BC. The study demonstrates that mutation in *FOXA1* unwinds the distinct chromatin profiles and influences therapeutic response in BC [67]. A well-established recent characterization study of PBX1 identified its importance as a valuable prognostic biomarker in BC. Defining signatures in chromatin facilitators involved in cancer characterized some of the pioneer factors like the GATA family of proteins, TLE1 protein, and PBX1 as a valuable prognostic biomarker in BC [68–70]. With the advent of newer technologies in genomics and proteomics, novel ER-associated pioneer factors have been continuously identified. Thus treatment targeting ER in conjugation with associated pioneer factors can effectively combat endocrine resistance for patients with BC.

## Concluding note and future perspectives

The study of the transcriptional apparatus and their associated proteins are continually renewing with advanced information available that can be used in various perspectives to target and regulate gene expression. TFs are an important class of regulatory proteins influencing the underlying molecular signaling leading to multiple malignancies. The molecular characterization of the deregulated TF and associated pathogenesis is of great concern and should also point to therapeutic approaches. Pioneer TF are an emerging class of hope towards the identification of deregulated mechanism and possible therapeutics. In the perspective study, we addressed the key pioneer factors involved in ER-dependent BC. However, only a few pioneer factors have been identified and studied in-depth, with high evidence collected from PTF, such as FOXA1 in prostate and BC, and GATA3 in ER-positive BC. Also, mechanistic details are only known for a small number of pioneer factors at present. Thus an elaborated study encompassing the role and potential therapeutic significance of PTF in BC can help identify predictive biomarkers in BC and later target with probable drug combinations. Future studies should address the relative efficacy of PTF targeted drugs in line, compared with anti-estrogen agents, based on the levels of expression of ER in BC, as well as their potential use as first-line therapy in selected ERα positive tumors, with low proliferating index, in combination with other targeted agents.

## Abbreviations

AR: androgen receptor
BC: breast cancer
ER: estrogen receptor
ERα: estrogen receptor α
ERβ: estrogen receptor β
FOXA1: forkhead box protein A1
GATA3: GATA binding protein 3
NF-κB: nuclear factor kappa-light-chain-enhancer of activated B cells
PBX1: pre-B-cell leukemia transcription factor 1
PTFs: pioneer transcription factors
TF: transcription factors
TLE1: transducin-like enhancer protein 1

## Declarations

### Copyright

© The Author(s) 2021.
